# Risk of Occult Contralateral Neck Metastasis in Early‐Stage HPV‐Related Lateralized Cancer of the Base of the Tongue

**DOI:** 10.1002/hed.70117

**Published:** 2025-12-10

**Authors:** Tyler R. Halle, Austin C. Cao, Anusha G. Naik, Gregory S. Weinstein, Jake J. Lee, Theodore A. Gobillot, Erin R. Kaye, Robert M. Brody, Devraj Basu, Bert W. O'Malley, D. Gregory Farwell, Steven B. Cannady, Alexander Lin, John Lukens, Michelle Gentile, Jason G. Newman, Ara A. Chalian, Christopher H. Rasskeh, Karthik Rajasekaran

**Affiliations:** ^1^ Department of Otolaryngology Emory University Atlanta Georgia USA; ^2^ Department of Otorhinolaryngology‐Head & Neck Surgery University of Pennsylvania Philadelphia Pennsylvania USA; ^3^ Department of Otolaryngology‐Head & Neck Surgery Stanford Medicine Stanford California USA; ^4^ Department of Otolaryngology University of Miami Miami Florida USA; ^5^ Department of Otolaryngology University of Maryland Baltimore Maryland USA; ^6^ Department of Radiation Oncology University of Pennsylvania Philadelphia Pennsylvania USA; ^7^ Department of Otolaryngology Medical Univesity of South Carolina Charleston South Carolina USA; ^8^ Leonard Davis Institute of Health Economics University of Pennsylvania Pennsylvania USA

**Keywords:** base‐of‐tongue, cervical lymph node metastasis, HPV‐related oropharyngeal squamous cell carcinoma, transoral robotic surgery

## Abstract

**Objectives:**

(1) To determine the incidence of occult contralateral cervical lymph node metastasis in patients with early‐stage HPV‐associated base‐of‐tongue (BOT) oropharyngeal squamous cell carcinoma (OPSCC) treated with primary surgery; and (2) to compare survival and recurrence in patients who did and did not undergo contralateral neck treatment.

**Background:**

Contralateral neck management in patients with early‐stage HPV+ OPSCC of the BOT treated surgically remains controversial. Despite lacking data, most patients receive surgery and/or radiation to the contralateral neck based on historical incidence of occult lymph node metastasis in OPSCC.

**Methods:**

A retrospective chart review of patients with AJCC 7th edition pT1‐2, pN0‐1 HPV+ BOT OPSCC undergoing transoral robotic BOT resection and ipsilateral neck dissection with adjuvant treatment was conducted. The incidence of occult contralateral nodal metastasis was assessed. Overall and disease‐free survival were compared between patients who did and did not undergo contralateral neck treatment.

**Results:**

Of 106 patients meeting inclusion criteria, 46 (43.3%) did not undergo treatment of the contralateral neck with radiation or lymphadenectomy, whereas 29 (27.4%) received radiation alone to the contralateral neck, and 31 (29.2%) underwent elective contralateral neck dissection without identification of occult metastasis in any case. Overall survival (HR: 0.95, 95% CI: 0.23–4.00) and disease‐free survival (HR: 1.43, CI: 0.55–3.71) did not significantly differ between patients who did and did not receive treatment to the contralateral neck.

**Conclusion:**

Risk of occult contralateral cervical lymph node metastasis in patients with early‐stage HPV‐associated BOT OPSCC treated with primary surgery was low, prompting consideration of forgoing contralateral neck treatment in these patients.

**Abbreviations**
BOT: base‐of‐tongue
CND: contralateral nodal disease
CRT: chemoradiotherapy
END: elective neck dissection
HPV+: HPV‐associated
OPSCC: oropharyngeal squamous cell carcinoma
RT: radiotherapy
SCC: squamous cell carcinoma
TORS: transoral robotic surgery

## 1 Introduction

Human papillomavirus‐associated (HPV+) oropharyngeal squamous cell carcinoma (OPSCC) is increasingly common, with HPV+ disease overtaking tobacco‐related as the most common subtype in many countries [1, 2, 3]. It typically involves the tonsils or base of tongue (BOT) and generally affects younger patients who lack many of the traditional risk factors for mucosal head & neck cancers like tobacco and alcohol use [4]. Despite a strong predilection for regional lymphatic spread, HPV+ OPSCC has a generally favorable prognosis [5]. Interest in mitigating long‐term treatment‐related toxicities without compromising oncologic outcomes drives ongoing efforts to de‐escalate therapy for this disease.

Transoral robotic surgery (TORS) for BOT cancers offers a less invasive option than traditional open surgical approaches and has similar survival and functional outcomes as primary concurrent chemotherapy and radiation [6]. Cervical lymphadenectomy ipsilateral to the primary tumor, which may be therapeutic or elective, is the standard of care in patients with OPSCC undergoing primary transoral surgery [7].

Management of the neck contralateral to the primary tumor remains controversial in patients with BOT cancers, particularly in those patients who undergo TORS with ipsilateral neck dissection and do not have an indication for adjuvant radiotherapy (RT) based on the surgical pathology. There is a lack of data to guide clinical decision‐making in this population for whom treatment of the contralateral neck involves an additional operation or radiation that might otherwise not be performed. The American Society for Radiation Oncology (ASTRO) recommends treatment of bilateral necks in all patients with BOT cancers as well as patients with tonsil cancers involving the tongue base [8]. However, guidelines from the National Comprehensive Cancer Network (NCCN) and American Society of Clinical Oncology (ASCO) are equivocal on the subject. NCCN guidelines state: “Tumors in the base of tongue, posterior pharyngeal wall, and soft palate require consideration of bilateral neck treatment as do tumors of the tonsil invading the tongue base” [9]. ASCO guidelines recommend bilateral neck dissection for “tumors that extend to the midline tongue base or palate or that involve the posterior oropharyngeal wall” without providing more specific guidance for BOT tumors that do not involve the midline [7].

Some authors have proposed that patients with HPV+ OPSCC of the BOT in whom adverse pathologic features (extranodal extension (ENE), more than one pathologic lymph node, a single pathologic node > 3 cm, perineural invasion (PNI), and lymphovascular invasion (LVI)) are not found upon resection of the primary tumor and ipsilateral nodal basin are appropriate for omission of surgery or radiation to the contralateral nodal basin [10, 11, 12]. This study's aims were to (1) determine the rate of occult node positivity in patients with early‐stage HPV+ BOT OPSCC upon dissection of the contralateral neck and (2) compare oncologic outcomes between the patients in the low‐risk population who received surgery or radiation to the contralateral nodal basin and those who did not.

## 2 Methods

Approval for this study was obtained from the Institutional Review Board of the University of Pennsylvania under IRB #826710.

### 2.1 Patient Selection

Consecutive patients with HPV+ BOT OPSCC who underwent primary surgical treatment consisting of TORS BOT resection and ipsilateral neck dissection between March 2007 and March 2022 were enrolled. Subjects with early‐stage BOT cancers, specifically defined in this study as pathologic T‐stage 1–2 (pT1–2) and pathologic N‐stage 0–1 (pN0–1) per the American Joint Commission on Cancer (AJCC) Staging Manual 7th Edition, were included [13]. HPV status was determined based on p16 immunohistochemistry. Only patients with lateralized tumors, at least 1 cm lateral to the midline, were included. This was determined during the initial direct laryngoscopy and confirmed with imaging. Patients with contralateral nodal disease on exam or imaging were excluded, as were patients who did not undergo ipsilateral neck dissection. Those with a prior history of head and neck cancer or head and neck radiation were also excluded.

Recommendations for adjuvant therapies were made based on the final surgical pathology and after review by the multidisciplinary tumor board. Adjuvant radiotherapy was recommended for patients with a single pathologic lymph node > 3 cm, multiple pathologic lymph nodes, pathologic T3 and T4 or perineural invasion. Adjuvant chemoradiotherapy (CRT) was recommended for those with five or more pathologic lymph nodes, positive margins or lymph node extranodal extension–(both micro and > 2 mm). Patients undergoing adjuvant CRT received cisplatin unless they had a medical contraindication to platinum‐based therapy.

### 2.2 Covariates

A retrospective review of electronic medical records was performed for patients who met criteria for inclusion. Demographic information collected includes age, sex, race, smoking history greater than 10 pack‐years, Eastern Cooperative Oncology Group (ECOG) performance status, and Charlson Comorbidity Index (CCI). Clinicopathologic factors reviewed include primary tumor subsite, clinical and pathologic TNM stages according to the AJCC 7th Edition, LVI, PNI, ENE, staging of neck dissection, and adjuvant therapies.

Recurrences were classified by the site(s) of failure as determined by imaging and/or biopsy, including primary site (local) recurrence, cervical lymph node (regional) recurrence, and recurrence in sites outside the head and neck (distant). Overall survival (OS) and disease‐free survival (DFS) were calculated from the date of surgery.

### 2.3 Statistical Analysis

Patients were stratified into two cohorts: (1) those who underwent contralateral neck dissection or radiation therapy, and (2) no contralateral neck therapy. Patient and clinicopathologic variables were compared between the cohorts using Pearson's Chi‐squared test, Fisher's exact test, and Kruskal–Wallis rank sum test, when appropriate. Survival analyses were performed using nonparametric Kaplan–Meier methods, and Cox proportional hazards were used to compare OS and DFS between subgroups of interest. All testing was performed with a two‐sided alpha level of 0.05 and/or 95% confidence interval (CI). Statistical analyses were performed using Rstudio Version 2023.06.1 + 524 [Rstudio].

## 3 Results

### 3.1 Baseline Demographics and Treatment Characteristics

**FIGURE 1:**
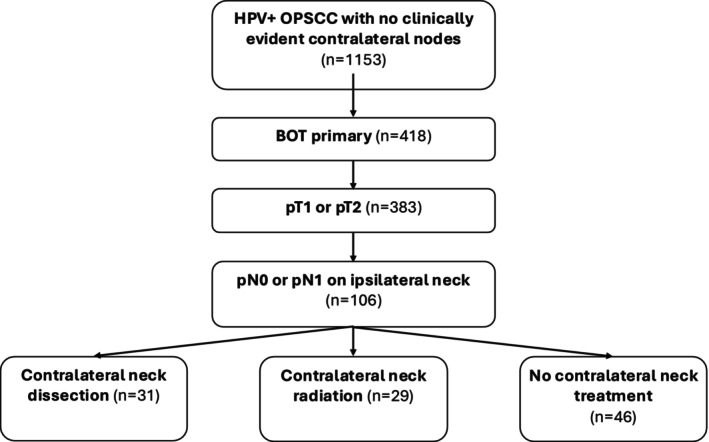
Flow chart illustrating patient selection and division into study cohorts.

Of 1127 patients with HPV+ OPSCC and no clinical contralateral nodal disease, 106 patients with treatment‐naïve AJCC 7th ed. pT1‐T2 pN0‐N1 HPV+ BOT SCC treated with TORS and ipsilateral neck dissection were included as demonstrated by the CONSORT diagram in Figure 1. This cohort was comprised of 46 patients (43.3%) who did not undergo any treatment of the contralateral neck with either radiation or neck dissection, 31 patients (29.2%) who underwent contralateral elective neck dissection (END), and 29 patients (27.4%) who received radiation to the contralateral neck. In total, 77 patients (72.6%) were managed with primary surgery as a single‐modality therapy.

There were no significant differences in the demographic characteristics between patients who had undergone treatment of the contralateral neck and those who had not (Table 1). The median age overall was 63 years (IQR: 57.5–68.5), and 81% of patients were male. There was no significant difference in smoking history, ECOG score, or CCI between these groups. Of the 29 patients who received adjuvant radiation to the contralateral neck, nine also required adjuvant chemotherapy based on pathologic staging.

**TABLE 1:** Patient and treatment characteristics.

Variable	Contralateral neck treatment, *N* = 60^a^	No contralateral neck treatment, *N* = 46^a^	*p* ^b^
Age	63 (12)	62 (11)	0.4
Sex			0.9
Male	49 (82%)	37 (80%)	
Female	11 (18%)	9 (20%)	
Race			0.7
White	55 (92%)	44 (96%)	
Non‐White	5 (8.3%)	2 (43%)	
Smoking history > 10 pack‐years	15 (25%)	15 (33%)	0.4
Charlton Comorbidity Index (CCI)			0.8
0	47 (78%)	34 (74%)	
1	7 (12%)	8 (17%)	
2 or greater	6 (10%)	4 (8.7%)	
Staged ipsilateral neck dissection	44 (73%)	**30 (65%)**	0.4
Neck dissection			< 0.001
Ipsilateral onIy	29 (48%)	46 (100%)	
Bilateral	31 (52%)	0 (0%)	
Adjuvant treatment			< 0.001
None	31 (52%)	46 (100%)	
Radiation	20 (33%)	0 (0%)	
Chemoradiation	9 (15%)	0 (0%)	

*Note*: Values shown in bold are statistically significant.

^a^ Median (IOR), *n* (%).

^b^ Wilcoxon rank sum Pearson's Chi‐squared test; Fisher's exact test.

### 3.2 Pathologic Characteristics and Incidence of Occult Contralateral Nodal Metastasis

Pathologic findings after primary surgical treatment are described in Table 2. Patients who received contralateral neck treatment had higher rates of regional metastasis (78% vs. 28%) and extranodal extension (15% vs. 0%). However, primary tumor characteristics such as tumor stage, lymphovascular invasion, and perineural invasion were not significantly different between the groups. Of note, three patients lacked LVI and PNI results due to complete removal of the primary after direct laryngoscopy and biopsy. Contralateral END was generally recommended to patients with BOT primaries who did not otherwise require adjuvant treatment based on their final surgical pathology (i.e., patients with pT1‐2 and pN0‐1 disease with no adverse features). Of the 31 patients that underwent contralateral END, occult cervical node metastasis was not found in any cases.

**TABLE 2:** Pathologic findings.

Variable	Contralateral neck treatment, *N* = 60^a^	No contralateral neck treatment, *N* = 46^a^	*p* ^b^
Pathologic N‐stage (AJCC 7th ed.)			**< 0.001**
p N0	13 (22%)	33 (72%)	
pN1	47 (78%)	13 (28%)	
Pathologic T‐stage (AJCC 7th ed.)			0.4
pT1	41 (68%)	28 (61%)	
pT2	19 (32%)	18 (39%)	
Lymphovascular invasion (LVI)	11 (19%)	3 (6.7%)	0.071
Perineural invasion {PNI)	7 (12%)	3 (6.7%)	0.5
Cervical level 4 or 5 involvement	1 (1.7%)	0 (0%)	> 0.9
Extranodal extension (ENE)	9 (15%)	0 (0%)	**0.005**
Number of ipsilateral lymph nodes examined	36 (14)	32 (16)	0.2
Contralateral neck involvement			**< 0.001**
Positive nodes on contralateral ND	0 (0%)	0 (0%)	
No positive nodes on contralateral ND	31 (52%)	0 (0%)	
Did not receive contralateral ND	29 (48%)	46 (100%)	

*Note*: Values shown in bold are statistically significant.

^a^ *n* (%); Median (IQR).

^b^ Pearson's Chi‐squared test; Fisher's exact test; Wilcoxon rank sum test.

### 3.3 Survival Analysis

The median follow‐up time was 49.1 months (IQR: 33.7–67.2 months). Among the 46 patients who did not undergo any treatment of the contralateral neck, two recurred locally, one recurred regionally in the ipsilateral neck, two recurred regionally in the contralateral neck, and one developed distant disease (Table 3). Of these, the two subjects with local recurrences had initial negative and close margins and recurred 1.2 and 2.9 years after initial primary tumor resection, respectively. The subject with ipsilateral neck recurrence recurred in level 5 and was treated with revision neck dissection and adjuvant radiotherapy. Of the two subjects with contralateral neck recurrences, one recurred in level 2 and was treated with neck dissection, and the second recurred in level 2 and was treated with neck dissection and adjuvant radiotherapy; both were able to be salvaged with no further evidence of disease through the follow‐up period.

**TABLE 3:** Site of first recurrence in patients who had received treatment to the contralateral neck compared to those who did not.

Characterisitic	Overall, *N* = 106^a^	Contralateral neck treatment, *N* = 60^a^	No contralateral neck treatment, *N* = 46^a^	*p* ^b^
Site of recurrence				0.2
Local	4 (3.8%)	2 (3.3%)	2 (4.3%)	
Regional (ipsllateral)	1 (0.9%)	0 (0%)	1 (2.2%)	
Regional (contralateral)	2 (1.9%)	0 (0%)	2 (4.3%)	
Distant	6 (5.7%)	5 (8.3%)	1 (2.2%)	
No recorded recurrence	93 (88%)	53 (88%)	40 (87%)	

^a^ *n* (%).

^b^ Fisher's exact test.

**FIGURE 2:**
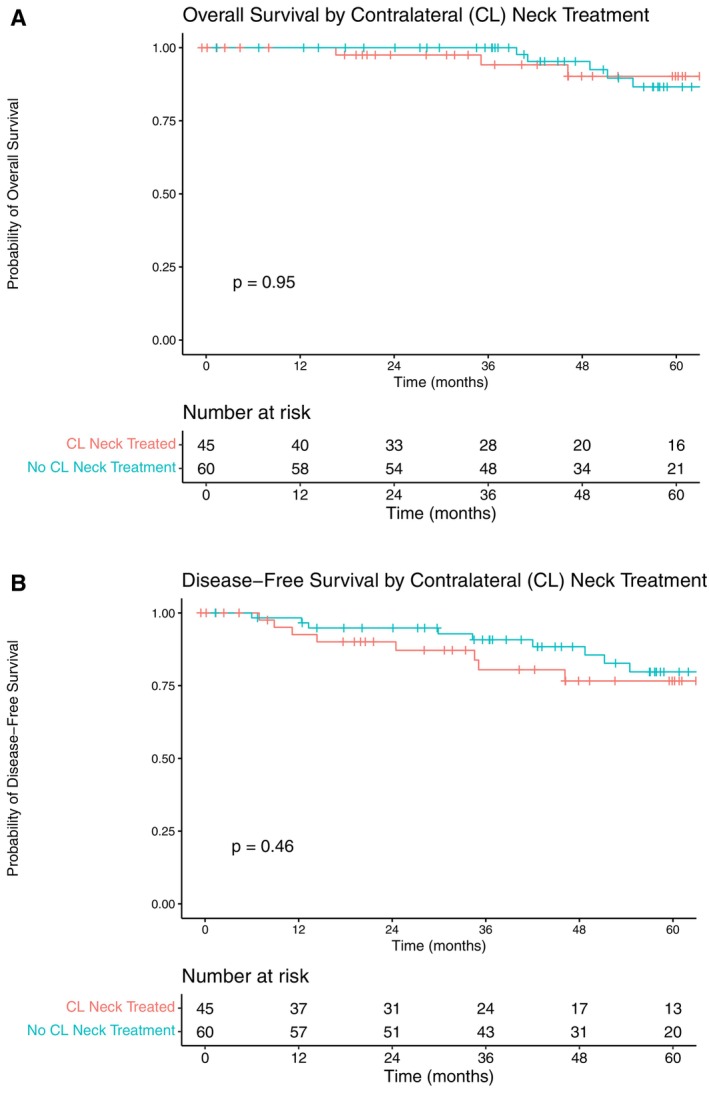
Overall survival (A) and disease‐free survival (B) of pN0‐1 patients. Those who received treatment of the contralateral neck are compared with those who did not. [Color figure can be viewed at wileyonlinelibrary.com]

When comparing those who received treatment of the contralateral neck to those who did not, there was no significant difference in overall survival (HR: 0.95, 95% CI: 0.23–4.00) or disease‐free survival (HR: 1.43, 0.55–3.71) (Figure 2).

## 4 Discussion

In this study of patients with pT1‐2 pN0‐1 HPV+ BOT SCC (AJCC 7th ed.) treated with primary TORS and ipsilateral cervical lymphadenectomy, occult contralateral lymph node metastasis was detected in none of the 31 patients who underwent bilateral neck dissection. The majority (72.6%) of patients were able to be treated with surgery only. We also found no differences in overall survival (OS) or disease‐free survival (DFS) among patients with pN0 or N1 disease based on whether they did or did not undergo contralateral neck treatment, consisting of either surgery or radiation therapy.

### 4.1 Risk of Contralateral Nodal Disease in Early‐Stage BOT Cancer

To our knowledge, this is the largest study to date investigating pathologic occult contralateral nodal disease (CND) in patients with early‐stage HPV‐associated lateralized BOT cancers treated with primary surgery. Previous studies have evaluated rates of CND in patients with OPSCC with incidence of occult CND ranging widely from zero to 33% [14]. However, many of these studies included patients with tonsil cancers, HPV‐ disease, and locally advanced (i.e., T3–T4) tumors, which limits their generalizability to patients with early‐stage cancers who may have the most to gain from treatment de‐intensification. At our institution, patients with smaller primary tumors and with no more than 1 lymph node involved, which must be ipsilateral to the primary tumor and < 3 cm without ENE if involved, are considered candidates for treatment de‐intensification and single‐modality treatment.

A recent meta‐analysis evaluated occult nodal disease in patients with OPSCC treated with primary surgery. In a sub‐analysis of patients with BOT cancers (*n* = 348), the incidence of occult CND was 0.68% in those with ipsilateral cN0 disease (*n* = 11) and 13.04% in those with ipsilateral cN1‐N2b disease (*n* = 84) (AJCC7) [14]. It is important to note that the authors did not distinguish between HPV+ and HPV‐ disease in this report. Other studies investigating differences in risk of contralateral nodal involvement between HPV+ and HPV‐ disease have yielded mixed results. Tritter et al. [15] found no difference in the incidence of contralateral nodal metastasis between 178 patients with HPV+ and HPV‐ disease. In contrast, a National Cancer Database study with over 15 000 patients with OPSCC found that HPV+ disease was weakly predictive of CND (OR 1.26, 99% confidence interval 1.10–1.44) [16]. Of note, both studies included a large proportion of patients with locally advanced (T3–4) primary tumors.

Among 120 patients with cT1‐2 HPV+ OPSCC treated with TORS and bilateral ND, Smith et al. [12] report a 0% incidence of pathologic CND among a subgroup of 6 patients with BOT cancers and cN0 (AJCC8) ipsilateral nodal disease. Among 54 individuals with cN1 disease, the incidence was 5.5%. When they excluded patients with BOT tumors involving the midline, 2 (4%) of 49 patients with cN0 or cN1 ipsilateral nodal disease had pathologic CND.

McMullen et al. [17] reported on contralateral nodal involvement in 32 patients with OPSCC, 75% of whom had HPV+ disease. They found occult CND was detected in 1 out of 19 patients (5.3%) with BOT cancers. Notably, this particular patient had an HPV‐ BOT cancer that involved the midline. The rate of occult CND was 7.4% (*n* = 2) among all patients (*n* = 27) and 5% (*n* = 1) in the p16+ cohort (*n* = 20), which included patients with tonsil and BOT tumors. Interestingly, the individual with HPV+ cancer associated with occult CND had a tonsil primary; none of the patients with HPV+ BOT cancers had occult CND. The authors also reported 33% sensitivity, 86% specificity, 93% negative predictive value, and 20% positive predictive value of preoperative imaging in identifying CND.

In one of the largest studies to date investigating occult CND in patients with HPV+ SCC of the BOT, Last et al. [11] found CND in 21.4% of 70 patients without preoperative evidence of CND on exam or imaging who underwent transoral surgery and bilateral neck dissection. The higher incidence of occult CND in that series compared to ours is likely due to the inclusion of patients with more advanced locoregional disease (18.6% had pT3‐4 tumors; 94.3% had N1‐2 disease [AJCC8]) as well as patients with disease involving the midline (37.1% had tumors crossing midline). Each of these features has been identified as risk factors for CND in multiple studies, though the relative impact of each is not clear [11, 12, 15, 16, 18, 19]. Of note, in the study by Last et al., the only risk factor specifically associated with occult CND was midline involvement.

Few studies have reported survival outcomes among patients with HPV+ tongue base SCC treated with primary transoral surgery. We found no difference in OS or DFS between patients with pN0‐1 (AJCC7) ipsilateral neck disease who received contralateral neck irradiation or neck dissection and those who did not undergo treatment of the contralateral neck. Favorable outcomes have also been reported in recent studies evaluating select patients with lateralized tumors who did not undergo contralateral neck treatment [10, 20]. Prior studies have also shown that patients with HPV+ OPSCC who do not undergo guideline‐recommended postoperative radiotherapy have an increased risk of locoregional failure but similar survival due to high salvage rates [21, 22]. Notably, the hazard ratio for disease‐free survival was high in our observational study but did not reach statistical significance. Further description of these outcomes in larger cohorts will be necessary to determine the best use cases for elective treatment of the contralateral neck.

In a recent study, Sahovaler et al. [20] evaluated survival and recurrence outcomes in 32 patients with well‐lateralized (defined as > 1 cm from midline) tonsil or BOT cancers treated with TORS and unilateral neck dissection and who did not undergo surgery or radiation to the contralateral neck. At 3 years, there were no contralateral neck failures. Local control and overall survival were both 96%, with one patient who developed a second primary and one patient who died of an unrelated rectal cancer.

Contreras et al. [10] evaluated recurrence and survival outcomes among 72 patients with mucosal head and neck cancers and cN0 contralateral necks who underwent primary surgical resection and neck dissection. Although patients with non‐oropharyngeal cancers were included, 51.4% of the sample had oropharyngeal primaries, and 86.5% of these were HPV‐associated. Of these, 71% of tumors involved midline structures. Postoperative radiation was delivered to the ipsilateral neck in patients with positive nodal disease but was excluded in 17/19 (89%) patients with pN0 ipsilateral neck disease. No patients included in the study received contralateral neck radiation. 5‐year regional control in the unirradiated neck was 97%; both regional recurrences in unirradiated necks occurred in patients with concomitant local recurrence. Five‐year rates of local control, progression‐free survival, and overall survival were 84%, 60%, and 64%, respectively.

Overall, our results and the current literature suggest that treatment of the contralateral neck may not be necessary in patients with early‐stage (pT1‐2 N0‐1 [AJCC7]) lateralized BOT cancers who are treated with primary transoral surgery and ipsilateral neck dissection. Although they represent a relatively small proportion of all patients with HPV+ OPSCC, this group is of particular interest because they comprise a distinct, low‐risk subset of patients with BOT primaries who may benefit from volume de‐intensification by omitting treatment of the contralateral neck.

### 4.2 Limitations

Our study has several limitations including the retrospective nature and associated potential for selection and observation biases. Patients with large tumors, tumors crossing the midline, and extensive nodal disease are generally poor surgical candidates and thus not well represented in studies like ours. This impacts generalizability. Additionally, the sample size of this study limited more detailed analyses of the subset of patients with recurrences, such as the analysis of any predisposing risk factors for recurrence. Because of the importance of pathologic data for accurate risk stratification, the contralateral neck should be managed cautiously in patients treated non‐surgically with primary chemoradiotherapy.

Our study also focuses on a small subset of particularly low‐risk patients. While patients with more advanced nodal disease may also benefit from volume de‐intensification by ipsilateral neck treatment alone, our study did not attempt to address this population. Emerging technologies may help to further stratify patients who are suitable for de‐intensification. For example, SPECT–CT for preoperative lymphatic mapping has recently shown early promise for identifying tumors with contralateral cervical nodal drainage who are at higher risk for occult CND [23]. HPV circulating tumor DNA may also have a role to play in identifying low‐risk patients suitable for de‐intensification [24].

## 5 Conclusions

The risk of occult contralateral nodal disease is very low in patients with early‐stage, lateralized HPV‐associated SCC of the BOT with pN0–N1 nodal disease. Treatment of the contralateral neck with surgery or radiation may be omitted in this population without compromising oncologic outcomes, with attention to the trend toward contralateral neck treatment in patients with pathologic ipsilateral nodal disease and/or extranodal extension in our study cohort.

## Disclosure

The authors have nothing to report.

## Conflicts of Interest

The authors declare no conflicts of interest.

## Data Availability

The data that support the findings of this study are available from the corresponding author upon reasonable request.
